# Study Protocol of a Pilot Trial Evaluating the Efficacy of an Integrated Therapeutic Intervention Based on Role-Playing Games (RPGs) in Adolescents and Young Adults with Anxiety, Depression and Emotional Dysregulation Disorders

**DOI:** 10.3390/brainsci16030281

**Published:** 2026-02-28

**Authors:** Cristiano Lupi, Laura Orsolini, Alberto Conte, Giuseppe Loris Nuzzo, Umberto Volpe

**Affiliations:** 1 Unit of Clinical Psychiatry, Department of Clinical Neurosciences/DIMSC, Polytechnic University of Marche, 60126 Ancona, Italy; cristiano.lupi89@gmail.com (C.L.); alberto.conte@pm.univpm.it (A.C.); giuseppelorisnuzzo97@gmail.com (G.L.N.); u.volpe@staff.univpm.it (U.V.); 2 Unit of Clinical Psychiatry, Department of Neurosciences/DIMSC, School of Medicine, Polytechnic University of Marche, 60126 Ancona, Italy

**Keywords:** adolescent mental health, youth anxiety, youth depression, emotional regulation, gaming, Role-Playing Games (RPGs)

## Abstract

Adolescence and early adulthood are critical developmental periods marked by an increasing vulnerability to emotional dysregulation and social difficulties, highlighting the need for engaging psychosocial interventions. This protocol presents a pilot study on the efficacy, feasibility, and acceptability of a structured group intervention based on Role-Playing Games (RPGs), designed to promote and support psychological well-being in transitional-aged youths. The study plans to recruit 54 participants (aged 15–24) who will take part in a 12 weekly, 2 h RPG-based intervention facilitated by trained clinicians. These clinicians will guide patients through narrative role-playing and a guided mentalization-based therapy through the gaming experience. All participants will be assessed at pre-, mid- and post-intervention, as well as during the 1- and 6-month follow-up, in the following dimensions: (a) mood, (b) anxiety, (c) emotional regulation, (d) alexithymia, and (e) coping skills. The following assessment tools will be administered: Hospital Anxiety and Depression Scale (HADS), Difficulties in Emotion Regulation Scale (DERS), Toronto Alexithymia Scale (TAS-20), and Coping Orientation to Problems Experienced (Brief-COPE). We expect the trial pilot will demonstrate good feasibility, greater participant engagement and treatment adherence, and improvements in all emotional and affective dimensions. This study seeks to establish foundational data to inform larger randomized controlled trials, with a follow-up, positioning RPG-based group interventions as potentially accessible, engaging, and convenient tools within youth mental health services.

## 1. Introduction

In an individual’s growth process, adolescence represents a delicate stage, from a neurobiological, psychological, and relational perspective [1]. Hence, it is not so surprising that most mental disorders have their onset during this period of life [2]. However, it is known that multiple factors can affect the psychological well-being of adolescents and young adults [2]. Exploring sexuality, experimenting themselves with new social roles, and comparing oneself with peers are some of the fundamental physiological experiences that can lead to possible difficulties in recognizing a new and more complex self-image, emotional distress, and, possibly, psychological suffering [3]. For instance, the constant engagement in daily activities, conflicts with peers, and family relationships are among the major stressors identified by adolescents [4]. A recent longitudinal analysis found that the prevalence of depressive symptoms among adolescents increased from 24% (95% CI: 0.19–0.28) between 2001 and 2010 to 37% (95% CI: 0.32–0.42) between 2011 and 2020 [5]. Recent studies found that approximately 75% of all mental disorders have an onset before age 24, often with unrecognized prodromal symptoms that have been present for even longer [2,6]. If these symptoms are not recognized and treated promptly, they can worsen and lead to the development of full-blown serious mental disorders as people age. For example, it is known that subjects experiencing depressive symptomatology during adolescence are at greater risk of developing depression and/or a recurrence of depressive symptomatology in adulthood [2,3,6]. Furthermore, young individuals with mental disorders have been demonstrated to manifest a significant impairment in the mentalization process (i.e., the ability to attribute mental states such as intentions, desires, emotions and knowledge to themselves and others), a competence defined as “Theory of Mind” (ToM) [7,8].

Therefore, taking into account the short- and long-term consequences associated with mental disorders, new intervention models are needed to promote integrated, multidisciplinary early prevention strategies [9]. Among the many psychological interventions proposed in recent decades, one of the most frequently identified tools for fostering more harmonious mental development in adolescence has been the therapeutic application of play. Historically, the Swiss psychologist Jean Piaget (1959) [10] already attributed a dual function to the play. According to Piaget’s theory, the play could act as both a developmental indicator (i.e., a direct expression of evolutionary processes) and as a developmental tool itself (“*play not only reflects development but actively contributes to it*”) (Piaget, 1959) [10]. Subsequently, within the psychoanalytic field, Winnicott (1971) also delved deeply into the potentially therapeutic function of play [11]. Hence, various authors identified one of the key strengths of the therapeutic value of the play in childhood and adolescence, such as the opportunity to actively explore aspects of the self, using resources that draw upon one’s own creativity and skills in facing complex situations [11]. It is through experimentation in a safe environment, such as that provided by play, that an individual’s growth process can be encouraged, training their relational and problem-solving abilities, ultimately leading to the modification of behaviors and habits, as well as the development of new resources [12].

Overall, several studies indicated that play may allow individuals to actively and safely self-explore themselves, by improving relational and problem-solving skills, modifying behaviors and habits, and promoting the development of new resources [12]. Among the various clinical applications of play, one innovative approach is represented by Role-Playing Games (RPGs), which can be delivered in both traditional “tabletop” and digital or online formats [13]. A RPG (Role-Playing Game) is a game in which players assume the specific role of one or more characters within an imagined space and narrative setting. Within this setting, fictional events occur, to which players react and interact with one another through a dialectical exchange [14]. To the standard fictional elements of a game, the RPG adds the specific element of “interpretation” or “role-playing.” As Bondioli (1994) [15] emphasized, this concerns the emotions and feelings of the participants more than the objective reality. The group and collaborative dynamics of RPGs encourage coping strategies, problem solving, and resocialization [16]. In a controlled study, RPG players scored significantly higher on the Interpersonal Reactivity Index (IRI) compared to non-players, supporting the hypothesis that role-players may exhibit a greater empathic engagement [17]. More recently, RPGs have been proposed as operational tools to enhance metacognitive abilities and ToM in clinical, educational, and community settings (Scicchitano, 2019) [18]. This evidence aligns with the growing body of research on gamified mental health interventions, which are designed to promote resilience, well-being, and treatment adherence through the use of game mechanics such as goal-setting and rewards [19,20,21]. Digital gamification strategies have been shown to improve motivation, perceived competence, and treatment adherence, while also reducing depressive symptoms in patients resistant to pharmacological treatments [22]. In particular, Serious Games have recently been introduced into transitional psychiatry. For example, an online RPG designed for individuals with a first episode of psychosis was found to enhance hope, empowerment, and social support while reducing social and self-directed stigma [23].

While these preliminary findings suggest that RPGs may serve as promising therapeutic and resocialization tools, especially in the early stages of youth mental disorders, the current body of experimental evidence remains limited [24,25]. Empirical validation of the use of RPGs in youths with anxiety, depression, and emotional dysregulation disorders is essential to confirm clinical utility in real-world youth mental health: rigorous assessment of feasibility, acceptability, and effectiveness when this therapeutic approach complements standard interventions, demonstrating measurable outcomes, practicality of implementation, stakeholder support, and lasting benefits. Such a comprehensive assessment, focused on new components and contexts, justifies its widespread implementation in practice. Further empirical validation is required to establish their clinical utility in real-world settings, by specifically assessing the feasibility, acceptability and efficacy of this therapeutic approach as complementary to treatment-as-usual interventions in youth mental health. Within this context, the current pilot study aims to evaluate the feasibility, acceptability and efficacy of an integrated therapeutic intervention based on RPGs in fostering more positive and better-regulated affect, as well as improving mood, anxiety, emotional self-regulation, coping strategies, illness insight, self-reflectiveness, and ToM abilities among transitional-aged adolescents and young adults consecutively recruited in a Transitional Psychiatry out/inpatient setting. The primary objective was to evaluate the efficacy of an integrated RPG-based intervention compared to the treatment-as-usual (TAU) in increasing positive affect (reducing anxiety and/or depressive symptomatology) and improving emotional regulation in adolescents and young adults. Secondary outcomes include: (a) assessing the acceptability and feasibility of the intervention within youths by comparing an in-person interventional setting versus an online setting; (b) evaluating the long-term efficacy during the follow-up at 1 and 6 months after the conclusion of the intervention. Exploratory objectives include evaluating the efficacy of the intervention in the improvement of coping strategies, illness insight, self-reflectiveness, and ToM abilities, compared to TAU.

## 2. Materials and Methods

### 2.1. Study Design

This pilot study was designed as a single-center, randomized controlled prospective interventional study. The intervention was developed to be addressed to a cohort of adolescents and young adults (aged 15–24) consecutively recruited at the Transitional Psychiatry service, Unit of Clinical Psychiatry, Department of Neurosciences/DIMSC, University Hospital of Marche Region within the Polytechnic University of Marche, Ancona, Italy. The randomization procedure will be performed using IBM Statistical Package for Social Sciences software, version 26 (SPSS, Chicago, IL, USA). For each participant, a random number will be generated using the RV.UNIFORM(0, 1) function, which produces values distributed uniformly between 0 and 1. The cases will be then sorted according to the random number obtained and assigned to the two groups (experimental versus TAU) in a 1:1 ratio. The allocation sequence will be generated by an independent researcher not involved in recruitment or intervention delivery, in order to ensure concealed allocation and minimize possible bias. The entire procedure will be documented to ensure replicability and methodological transparency. The participants of the experimental group will also be randomly divided into either an in-person or remote group setting.

### 2.2. Participants and Recruitment

Patients referred to the Transition Psychiatry service will be invited to participate in the study if they meet the following inclusion criteria: (a) diagnosis of an emotional and/or affective disorder (i.e., Major Depressive Disorder, Bipolar Disorder, Cyclothymia, Persistent Depressive Disorder, Seasonal Affective Disorder, Disruptive Mood Dysregulation Disorder, Other Specified Depressive Disorder, Unspecified Depressive Disorder, Other Specified Bipolar and Related Disorder, Unspecified Bipolar and Related Disorder, Unspecified Mood Disorder, Oppositional Defiant Disorder, Intermittent Explosive Disorder, Conduct Disorder, Other Specified Disruptive, Impulse-Control, and Conduct Disorder, Unspecified Disruptive, Impulse-Control, and Conduct Disorder) according to the *Diagnostic and Statistical Manual of Mental Disorders, Fifth Edition* text revised (DSM-5-TR) (APA, 2022) [26], following a semi-structured clinical interview conducted by a senior specialized psychiatrist of the outpatient Transition Psychiatry ward; (b) aged 15–24 years old; (c) a score of ≥8 at the HADS (Hospital Anxiety and Depression Scale) depression (HADS-D) and anxiety (HADS-A) subscales [27,28]; (d) a score of ≥ 95 at the DERS (Difficulties in Emotion Regulation Scale) [29,30,31]; (e) agreement to the study participation; (f) ability to provide written informed consent (their parents, in the case they are less than 18 years old). Exclusion criteria include the following: (a) current psychotic symptomatology (i.e., delusions, hallucinations, disorganized speech or behavior), which may limit the adherence and intervention participation; (b) intellectual disability and/or mental retardation and/or difficulty in understanding Italian language and/or learning and/or moderate-to-severe cognitive deficits (absolute contraindication); (c) sensory or communication deficits that could interfere with the study participation; (d) current substance and/or alcohol use (including acute and chronic intoxication, and recent substance and/or alcohol intake during the last three months); (e) current mania and/or manic states; (f) comorbidity with personality disorder. All included participants will continue to receive the treatments usually provided (e.g., regular psychiatric visits, pharmacological treatments, and psychotherapy) (TAU).

The study will be conducted in accordance with the principles of the Declaration of Helsinki and in compliance with current regulations on the protection of personal data (Legislative Decree 196/03, Italian Personal Data Protection Code). All participants (or their legal guardians, in the case of participants aged lower than 18-years-old) will provide written informed consent, after full description of study protocol. The study investigators will ensure that all mental health professionals involved in the study are qualified and informed about the protocol, interventions, and trial-related duties. Data will be collected anonymously and stored in password-protected databases, accessible only to the researchers involved in the study. The study protocol has been approved by the Institutional Ethical Committee of Marche Region (Prot. 201/2024).

### 2.3. Assessment Tools and Measurements

Assessments will be carried out at baseline (t0—pre-intervention), at 1 month (t1), at 3 months (t2—post-intervention), and during a follow-up at 1 month and 6 months after the conclusion of the intervention. Administered at baseline will be an ad hoc case-report form to collect the socio-demographic and clinical data, including age, sex, nationality, educational level, marital status, living and occupational condition, as well as medical and pharmacological history (i.e., age of illness onset, current and/or previous pharmacological and/or not pharmacological therapy, previous hospitalizations in psychiatric ward and/or medical settings, suicidality history, substance and/or alcohol use history). At the assessment time points, all participants will be administered the following assessment tools: (a) HADS [27,28]; (b) DERS [29,30]; (c) Toronto Alexithymia Scale (TAS-20) [32,33]; and (d) Coping Orientation to Problems Experienced (Brief-COPE) [34].

The Italian version of the HADS is a 14-item self-report measure able to assess the anxiety and depression levels of participants [28]. The HADS is constituted by seven items relating to anxiety (anxiety subscale) and seven items relating to depression (depression subscale). Items are answered in a four-point response format with a total score ranging from 0 to 21 for each of the two subscales: the higher the score, the more severe the anxiety or depression. The Italian version of the DERS [30,35] is a 36-item self-report questionnaire on a five-point Likert scale assessing the level of difficulties in emotional regulation. The DERS contains a total score (ranging from 6 to 180) and six subscales assessing the following domains: (a) non-acceptance of negative emotions; (b) inability to engage in goal-directed behaviors when experiencing negative emotions; (c) difficulty in controlling impulsive behaviors when experiencing negative emotions; (d) limited access to effective emotion regulation strategies; (e) lack of own emotional awareness and clarity. The TAS-20 is the most common tool for assessing alexithymia. It is composed of 20 items, and each item is graded according to a five-point Likert-type scale ranging from 1 = “strongly disagree” to 5 = “strongly agree”. Total scores range from 20 to 100, with a score ≥ of 61 indicative of alexithymia and scores between 51 and 60 of borderline alexithymia. The TAS-20 has a three-factor structure: F1, difficulty in identifying feelings; F2, difficulty in describing feelings to others; F3, externally oriented thinking style. The Italian version of the questionnaire’s internal consistency is good (Cronbach’s alpha = 0.81) and has the same structure as the original English version [32,33]. The Brief-COPE is a 28-item abbreviated version of the original 60-item COPE Inventory [34,36]. The scale assesses the primary coping styles on three subscales: (a) problem-focused coping, characterized by active coping, use of informational support, planning, and positive reframing; (b) emotion-focused coping, characterized by venting, emotional support seeking, humor, acceptance, self-blame, and religion; (c) avoidant coping strategies, characterized by self-distraction, denial, substance use, and behavioral disengagement. In addition to these three subscales, the Brief-COPE also provides scores for 14 individual facets: self-distraction, denial, substance use, behavioral disengagement, emotional support, venting, humor, acceptance, self-blame, religion, active coping, use of instrumental support, positive reframing, and planning.

At the end of intervention, participants will be asked to fill out two specifically designed questionnaires to assess the feasibility and acceptability of the intervention, both in-person versus online (Supplementary Materials). These data will be gathered through both qualitative and semi-quantitative measures, consisting of open-ended questions and items rated on a Likert scale.

### 2.4. Statistical Analysis

The IBM Statistical Package for Social Sciences software, version 26 (SPSS, Chicago, IL, USA), will be used for the data analysis. Sample size has been calculated using the Statistical Software G*Power version 3.1. (Franz, Universitat Kiel, Kiel, Germany). Based on the primary objective of the study, to detect any difference in the HADS mean scores in both interventional groups (online versus face-to-face) and between TAU versus TAU + interventional group at t0 (pre-intervention) and t2 (post-intervention), and 1 month (t3) and 6 months (t4) follow-up, a statistical power of 0.80 will be established to identify an effect size of 0.5 (α = 0.05, two-tailed), taking into consideration all variables to be entered in the repeated-measures *t*-test (Zα/2 = 1.96, Zβ = 0.84, σ = 5 as expected standard deviation in the HADS scores, Δ = 3; N = (1.96 + 0.84)^2^ × 2 × 52 = 7.84 × 50 ≈ 44). Therefore, considering a possible 20% dropout rate, a total sample size of 54 was established to be reached for the present study (27 participants for each group). Descriptive analyses will be performed to describe the socio-demographic characteristics of the sample and to assess the normality of the data. Categorical variables will be summarized as frequency (n) and percentage (%), while continuous variables will be summarized as mean and standard deviation (SD). Categorical variables will also be compared using the χ^2^-test and post hoc tests. To summarize the data and test the differences based on categorical analysis, parametric tests (*t*-test or ANOVA) or non-parametric tests (Kruskal–Wallis test or Mann–Whitney U test) will be used, depending on the normality of the data. Data will be analyzed for inferential statistics by using mixed-effects models to analyze the temporal trajectories of the outcome variables at different time points (t0–t4). Coefficients obtained by the regression models will be compared to evaluate the efficacy of the intervention depending on in-person versus online modality. Repeated-measures ANOVA corrected for sphericity (Mauchly’s test and Greenhouse–Geisser correction, if necessary) will be carried out. Correlation and regression analyses (univariate/multivariate, linear/logistic) will be conducted, including socio-demographic and clinical variables and the total scores and subscales of all primary and secondary outcomes, to explore any relationships between the different factors. The statistical significance cut-off will be set at *p* < 0.05.

## 3. Experimental Intervention

The intervention utilizes the open-source Basic Fantasy RPG ruleset (https://www.basicfantasy.org/ (accessed on 31 May 2025)) and a narrative storyline specifically developed for therapeutic purposes. The groups will each consist of five participants, led by a “Game Master” and a designated Observer. Both Game Master and Observer are represented by trained clinicians. The Game Master will be responsible for managing the rules and dynamics of the game, while the Observer will be responsible for recording the participants’ communication (e.g., verbal and non-verbal) variables as well as the participants’ interpersonal and relational dynamics during the session. The intervention will be randomly provided on-site (face-to-face) or by using online modality.

### 3.1. Experimental Intervention On-Site (Face-to-Face)

During the first session, participants will be stimulated to present themselves to each group participant, the Game Master and the Observer will describe the intervention, and the setting, rules, and characters of the story will be assigned and defined for the next sessions of the RPG-based intervention. Before starting the intervention, the Game Master and the Observer will provide the details regarding the specific topics that could be elicited during the intervention, creating an inclusive and respectful gaming environment that is acceptable to all participants. The opportunity to address specific sensitive topics/issues related to family dynamics, interpersonal relationships, personal experiences, gender-sensitive and cultural aspects will be explored during this pre-intervention session. This preliminary evaluation will allow clinicians to preliminarily identify sensitive topics and provide a management plan to participants during the interventional study. In this manner, the intent is to create a peaceful and pleasant atmosphere, in which participants can feel free to express themselves and enjoy the gaming experience in a constructive and effective way.

The program includes 12 weekly sessions, extending over a period of three months. Each session will last approximately 120 min and will be structured in two phases: (1) a gaming phase (90 min), a collaborative gaming session set in a medieval fantasy context; and (2) a metacognitive phase (30 min), a guided reflection on the gaming events, focusing on working memory, identification of mental states, cognitive decentering, and the transfer of acquired skills to real-life situations.

The metacognitive phase will comprise four steps, as detailed below:

(1) Reflection on the gaming experience: After completing the role-playing session, the Game Master and the Observer will guide the group in reflecting on the gaming experience. The cognitive skill to be developed during this task will be the working memory. Participants will be asked to recall and describe the events of the experienced game and the characters’ actions and behaviors during the play. This ability will be measured by observing how effectively participants correctly recall the events of the game.
(2) Identification of mental states: The Game Master and the Observer will help participants to identify their own mental states and those of the game characters. The cognitive skills to be developed are represented by ToM. Participants will be asked to remember, describe, and understand their own thoughts, emotions, and motivations and those of the game characters. This skill will be measured by observing how effectively participants perform this task.
(3) Exploration of participants’ perspectives: The Game Master and the Observer will encourage participants to explore the different perspectives of the game characters. The cognitive skill to be developed is represented by cognitive decentralization. Participants will be encouraged to take the perspective of other characters in addition to their own and observe the events and the story from other characters’ point of view. This skill will be measured by observing how effectively the participants perform this task.
(4) The connection with real-life experiences: The Game Master and the Observer will help participants connect the role-playing experience with their own lives. The cognitive skill to be developed is represented by the life transfer. Participants will be encouraged to connect the skills learned during the gaming experience with their own lives. This skill will be measured by observing how effectively participants perform this task.

### 3.2. Experimental Intervention Online

For the online setting, all sessions will be conducted via Microsoft Teams, with the exception of the first session, which will be held in person to facilitate group cohesion, with the same characteristics described for the experimental intervention on-site. The number, duration and characteristics of each session will be the same as illustrated in the experimental on-site intervention.

## 4. Discussion

This protocol describes a randomized pilot single-center clinical trial aiming at assessing the feasibility, acceptability and efficacy of an integrated RPG-based intervention for adolescents and young adults with emotional and/or affective disorders, recruited at a Transitional Psychiatry service specifically addressing to 15–24-aged patients. The study introduces an approach using RPGs as an integrative intervention to TAU for adolescents and young outpatients, by comparing an on-site (face-to-face) versus an online delivery modality. The primary outcome is evaluating the efficacy in the pre-/post-interventions in terms of improvement of anxiety and/or depressive symptomatology, as well as emotional regulation strategies. The secondary outcomes have been established to investigate any significant differences in the efficacy, acceptability, and feasibility by comparing online versus face-to-face modality, as well as investigating other psychopathological dimensions (such as alexithymia, coping strategies, ToM abilities). The exploratory outcome includes evaluating the maintenance of intervention efficacy through the time, during the 1 month and 6 month post-intervention follow-up period.

The intervention design is able to provide a distinctive element in the combination of an experiential phase (“Gaming Phase”) and a reflective phase (“Metacognitive Phase”), which respectively allow activation of gaming dynamics and subsequent metacognitive elaboration. This structure is consistent with theoretical and experimental evidence recognizing the usefulness of play (in a broader sense) in developing relational skills, Theory of Mind, and coping strategies [16,37,38]. Another strength of the study is represented by the dual delivery modality, in-person and online. The latter aligns with recent digital innovations in mental health, already tested in other psychosocial protocols, and allows for overcoming logistical barriers, ensuring greater accessibility for a youth population often more inclined to digital interaction [39,40,41,42,43,44,45].

### 4.1. Limitations of the Study and Future Research Directions

Despite the study’s potential strong points, it may have some limitations. Firstly, this is a pilot trial with a limited sample size that may restrict the statistical power and the generalizability of the results. Secondly, the recruitment of participants from a single clinical center reduces the sample’s heterogeneity and may limit the generalizability of the findings. A further critical point concerns the potential influence of confounding factors, such as the coexistence of pharmacological treatments, the variability of clinical symptomatology, or the differing levels of patient familiarity with gaming. Finally, while the comparison between in-person and remote settings is methodologically interesting, it will require caution in data interpretation, as relational dynamics may differ significantly between the two contexts. However, the longitudinal study design and the advantage in recruiting participants to be randomly allocated to two different settings could help clinicians in further developing larger and multicenter studies able to manage these confounding variables and more focused on on-site versus online modality. If our findings confirm the expected efficacy of the RPG-based intervention in youth mental health, the protocol could be developed as a model to be replicated in other clinical centers. Based on the study findings, future studies should include larger, multicenter samples to increase the external validity of the results. It will also be important to investigate the underlying mechanisms of change responsible for the efficacy of RPGs, with a particular focus on the role of group dynamics and the ability to transfer acquired skills to real-life contexts. Finally, exploring adaptations of the protocol for specific clinical conditions (e.g., anxiety disorders, mood disorders, conduct disorders) and/or integrating it with other evidence-based interventions could strengthen the impact of RPGs as an innovative, versatile, and culturally adaptable therapeutic tool.

### 4.2. Clinical Implications of the Study

Furthermore, the target population (adolescents and young adults) could help clinicians and researchers establish if future development of the integrated intervention should be carried out on-site and/or by online delivery, which would optimize human resources and increase the access to youth mental health services by patients who are more reluctant concerning face-to-face interventions. Demonstrating that there is an equal efficacy of the RPG-based intervention both online versus face-to-face could allow clinicians to recommend both modalities in clinical practice, depending on the level of psychopathology, perceived stigma, logistical issues and in-person engagement of the patient. The online modality could also represent a youth-friendly and stigma-free approach, capable in reaching a larger audience of adolescents and young adults.

## 5. Conclusions

Overall, if the findings demonstrate good acceptability, feasibility, and participants’ engagement to the RPG-based intervention, as well as efficacy in terms of improving anxiety and/or depressive symptomatology and/or emotional regulation difficulties in a cohort of adolescents and young adults, then the RPG-based approach could represent a valuable complementary therapeutic option within transitional psychiatry services, helping to bridge the gap in non-pharmacological interventions specifically designed for this age group. The emphasis placed on emotional regulation, the development of ToM abilities, and the resocialization process activated by the gaming experience within a therapeutic group, appears particularly relevant during a developmental period at high risk for psychopathological onset. Furthermore, the ability to deliver the intervention in a digital format creates interesting prospects in terms of scalability and sustainability, with potential benefits including reduced costs and a broadened target audience. In line with the most recent trends in digital mental health, RPGs, with their elements of gamification, may be capable of increasing patient engagement and treatment adherence.

## Data Availability

No new data were created or analyzed in this study.

## Supplementary Materials

The following supporting information can be downloaded at: https://www.mdpi.com/article/10.3390/brainsci16030281/s1, Supplementary Material S1: Satisfaction Questionnaire.

## References

[1] Hoffman E.A., LeBlanc K., Weiss S.R.B., Dowling G.J. (2022). Transforming the Future of Adolescent Health: Opportunities From the Adolescent Brain Cognitive Development Study. J. Adolesc. Health.

[2] Solmi M., Radua J., Olivola M., Croce E., Soardo L., de Pablo G.S., Shin J.I., Kirkbride J.B., Jones P., Kim J.H. (2022). Age at Onset of Mental Disorders Worldwide: Large-Scale Meta-Analysis of 192 Epidemiological Studies. Mol. Psychiatry.

[3] Racine N., McArthur B.A., Cooke J.E., Eirich R., Zhu J., Madigan S. (2021). Global Prevalence of Depressive and Anxiety Symptoms in Children and Adolescents during COVID-19: A Meta-Analysis. JAMA Pediatr..

[4] Kim K.M. (2021). What Makes Adolescents Psychologically Distressed? Life Events as Risk Factors for Depression and Suicide. Eur. Child Adolesc. Psychiatry.

[5] Shorey S., Ng E.D., Wong C.H.J. (2022). Global Prevalence of Depression and Elevated Depressive Symptoms among Adolescents: A Systematic Review and Meta-Analysis. Br. J. Clin. Psychol..

[6] Fusar-Poli P. (2019). Integrated Mental Health Services for the Developmental Period (0 to 25 Years): A Critical Review of the Evidence. Front. Psychiatry.

[7] Premack D., Woodruff G. (1978). Does the Chimpanzee Have a Theory of Mind?. Behav. Brain Sci..

[8] van Neerven T., Bos D.J., van Haren N.E.M. (2021). Deficiencies in Theory of Mind in Patients with Schizophrenia, Bipolar Disorder, and Major Depressive Disorder: A Systematic Review of Secondary Literature. Neurosci. Biobehav. Rev..

[9] Ortuño-Sierra J., Lucas-Molina B., Inchausti F., Fonseca-Pedrero E. (2021). Special Issue on Mental Health and Well-Being in Adolescence: Environment and Behavior. Int. J. Environ. Res. Public Health.

[10] Piaget J., Paul K. (1959). The Language and Thought of the Child.

[11] Hugh-Jones S., Ray S., Wilding A., Sutton M., Humphrey N., Munford L. (2025). Does Regular Engagement with Arts and Creative Activities Improve Adolescent Mental Health and Wellbeing? A Systematic Review and Assessment of Causality. SSM Popul. Health.

[12] Abbott M.S., Stauss K.A., Burnett A.F. (2022). Table-Top Role-Playing Games as a Therapeutic Intervention with Adults to Increase Social Connectedness. Soc. Work Groups.

[13] Yuliawati L., Wardhani P.A.P., Ng J.H. (2024). A Scoping Review of Tabletop Role-Playing Game (TTRPG) as Psychological Intervention: Potential Benefits and Future Directions. Psychol. Res. Behav. Manag..

[14] Grouling Cover J. (2010). The Creation of Narrative in Tabletop Role-Playing Games.

[15] Bondioli A., Savio D. (1994). Osservare il Gioco di Finzione: Una Scala di Valutazione delle Abilità Ludico-Simboliche Infantile (SVALSI).

[16] Kroplewski Z., Łoś R., Pawlicki B.J. (2025). Impact of Participation in Role-Playing Game (RPG) Sessions on the Perceived Level of Social Anxiety and Received Social Support. Brain Sci..

[17] Rivers A., Wickramasekera I.E., Pekala R.J., Rivers J.A. (2016). Empathic Features and Absorption in Fantasy Role-Playing. Am. J. Clin. Hypn..

[18] Scicchitano V. Role-Playing Games, Narrative Processes, and Metacognition in Clinical and Training Contexts. Proceedings of the Systemic-Relational Psychotherapy Conference.

[19] Litvin S., Saunders R., Maier M.A., Lüttke S. (2020). Gamification as an Approach to Improve Resilience and Reduce Attrition in Mobile Mental Health Interventions: A Randomized Controlled Trial. PLoS ONE.

[20] Cheng C., Ebrahimi O.V. (2023). Gamification: A Novel Approach to Mental Health Promotion. Curr. Psychiatry Rep..

[21] Sardi L., Idri A., Fernández-Alemán J.L. (2017). A Systematic Review of Gamification in E-Health. J. Biomed. Inform..

[22] Cheng V.W.S. (2020). Recommendations for Implementing Gamification for Mental Health and Wellbeing. Front. Psychol..

[23] Jankowski S., Ferreira K., Mascayano F., Donovan E., Rahim R., Birnbaum M.L., Yum-Chan S., Medoff D., Marcogliese B., Fang L. (2022). A Serious Game for Young People With First Episode Psychosis (OnTrack>The Game): Qualitative Findings of a Randomized Controlled Trial. JMIR Ment. Health.

[24] Battles A.R., Curland R.A., Cruitt P.J. (2025). A Pilot Evaluation of a Therapeutically Applied Tabletop Role Playing Game Group Therapy Among Veterans. Int. J. Group Psychother..

[25] Billieux J., Fournier L., Rochat L., Georgieva I., Eben C., Andersen M.M., King D.L., Simon O., Khazaal Y., Lieberoth A. (2025). Can Playing Dungeons and Dragons Be Good for You? A Registered Exploratory Pilot Programme Using Offline Tabletop Role-Playing Games to Mitigate Social Anxiety and Reduce Problematic Involvement in Multiplayer Online Video Games. R. Soc. Open Sci..

[26] American Psychiatric Association (2022). Diagnostic and Statistical Manual of Mental Disorders.

[27] Zigmond A.S., Snaith R.P. (1983). The Hospital Anxiety and Depression Scale. Acta Psychiatr. Scand..

[28] Costantini M., Musso M., Viterbori P., Bonci F., Del Mastro L., Garrone O., Venturini M., Morasso G. (1999). Detecting Psychological Distress in Cancer Patients: Validity of the Italian Version of the Hospital Anxiety and Depression Scale. Support. Care Cancer.

[29] Gratz K.L., Roemer L. (2004). Multidimensional Assessment of Emotion Regulation and Dysregulation: Development, Factor Structure, and Initial Validation of the Difficulties in Emotion Regulation Scale. J. Psychopathol. Behav. Assess..

[30] Giromini L., Velotti P., de Campora G., Bonalume L., Zavattini G.C. (2012). Cultural Adaptation of the Difficulties in Emotion Regulation Scale: Reliability and Validity of an Italian Version. J. Clin. Psychol..

[31] Erez C., Gordon I. (2025). The Imperfect Yet Valuable Difficulties in Emotion Regulation Scale: Factor Structure, Dimensionality, and Possible Cutoff Score. Assessment.

[32] Bagby R.M., Parker J.D.A., Taylor G.J. (1994). The Twenty-Item Toronto Alexithymia Scale-I. Item Selection and Cross-Validation of the Factor Structure. J. Psychosom. Res..

[33] Bressi C., Taylor G., Parker J., Bressi S., Brambilla V., Aguglia E., Allegranti I., Bongiorno A., Giberti F., Bucca M. (1996). Cross Validation of the Factor Structure of the 20-Item Toronto Alexithymia Scale: An Italian Multicenter Study. J. Psychosom. Res..

[34] Carver C.S. (1997). You Want to Measure Coping but Your Protocol’s Too Long: Consider the Brief COPE. Int. J. Behav. Med..

[35] Sighinolfi C. (2010). NPACLMISC Traduzione e Adattamento Italiano Del Difficulties in Emotion Regulation Strategies (DERS): Una Ricerca Preliminare. Psicoter. Cogn. Comport..

[36] Carver C.S., Scheier M.F., Weintraub K.J. (1989). Assessing Coping Strategies: A Theoretically Based Approach. J. Pers. Soc. Psychol..

[37] Henning G., de Oliveira R.R., de Andrade M.T.P., Gallo R.V., Benevides R.R., Gomes R.A.F., Fukue L.E.K., Lima A.V., de Oliveira M.B.B.Z.N., de Oliveira D.A.M. (2023). Social Skills Training with a Tabletop Role-Playing Game, before and during the Pandemic of 2020: In-Person and Online Group Sessions. Front. Psychiatry.

[38] Baker I.S., Turner I.J., Kotera Y. (2023). Role-Play Games (RPGs) for Mental Health (Why Not?): Roll for Initiative. Int. J. Ment. Health Addict..

[39] Kahl B.L., Miller H.M., Cairns K., Giniunas H., Nicholas M. (2020). Evaluation of ReachOut.Com, an Unstructured Digital Youth Mental Health Intervention: Prospective Cohort Study. JMIR Ment. Health.

[40] Orsolini L., Pompili S., Salvi V., Volpe U. (2021). A Systematic Review on Telemental Health in Youth Mental Health: Focus on Anxiety, Depression and Obsessive-Compulsive Disorder. Medicina.

[41] Orsolini L., Appignanesi C., Pompili S., Volpe U. (2022). The Role of Digital Tools in Providing Youth Mental Health: Results from an International Multi-Center Study. Int. Rev. Psychiatry.

[42] Orsolini L., Longo G., Volpe U. (2024). Practical Application of Digital Therapeutics in People with Mood Disorders. Curr. Opin. Psychiatry.

[43] Orsolini L., Longo G., Volpe U. (2025). Psychosocial Interventions in the Rehabilitation and the Management of Psychosis and Schizophrenia: A Systematic Review on Digitally-Delivered Interventions. Actas Esp. Psiquiatr..

[44] Cross S., Nicholas J., Mangelsdorf S., Valentine L., Baker S., McGorry P., Gleeson J., Alvarez-Jimenez M. (2023). Developing a Theory of Change for a Digital Youth Mental Health Service (Moderated Online Social Therapy): Mixed Methods Knowledge Synthesis Study. JMIR Form. Res..

[45] Alvarez-Jimenez M., Nicholas J., Valentine L., Liu P., Mangelsdorf S., Baker S., Gilbertson T., O’Loughlin G., McEnery C., McGorry P.D. (2025). A National Evaluation of a Multi-Modal, Blended, Digital Intervention Integrated within Australian Youth Mental Health Services. Acta Psychiatr. Scand..

