# Supplementary material for: Study Protocol of a Pilot Trial Evaluating the Efficacy of an Integrated Therapeutic Intervention Based on Role-Playing Games (RPGs) in Adolescents and Young Adults with Anxiety, Depression and Emotional Dysregulation Disorders
Source: Brain Sci. 2026 Feb 28;16(3):281. doi: 10.3390/brainsci16030281 (PMC13023783; doi:10.3390/brainsci16030281)
Supplement: Supplementary file 1 [file brainsci-16-00281-s001.zip › brainsci-4160903-supplementary.pdf]

END-OF-TREATMENT QUESTIONNAIRE

How was your overall satisfaction with this activity? Did you like it?

(Not at all) (Not very much) (Sufficiently) (A lot) (Very much)

Do you think that it was helpful?

(Not at all) (Not very much) (Sufficiently) (A lot) (Very much)

For what?

____

____

Did it meet your expectations or was it different?

(Not at all) (Not very much) (Sufficiently) (A lot) (Very much)

In what?

____

____

What was the element you appreciated the most?

____

____

Which is the thing you disliked or liked the least?

____

____

Did the role-playing sessions help you understand your emotions?

(Not at all) (Not very much) (Sufficiently) (A lot) (Very much)

Did the role-playing sessions help you understand the motivations behind your behavior and that of others?

(Not at all) (Not very much) (Sufficiently) (A lot) (Very much)

How do you feel about the conclusion of this study?

How do you rate the length of the role-playing sessions?

(Too short) (Short) (Adequate) (Long) (Too long)

Why?

How do you rate the length of the mentalization sessions?

(Too short) (Short) (Adequate) (Long) (Too long)

Why?

How do you rate the duration (12 sessions) of the treatment?

(Too short) (Short) (Adequate) (Long) (Too long)

Why?

Add a personal comment on an aspect of your choice that has not already been investigated by this questionnaire.
